# Cavernous hemangioma of the third ventricle: a case report and review of the literature

**DOI:** 10.1186/1477-7819-12-237

**Published:** 2014-07-29

**Authors:** Moon-Soo Han, Kyung-Sub Moon, Kyung-Hwa Lee, Seul-Kee Kim, Shin Jung

**Affiliations:** 1 Department of Nueurosurgery, Chonnam National University Research Institute of Medical Science, Chonnam National University Hwasun Hospital & Medical School, 322 Seoyang-ro, Hwasun-eup, Hwasun-gun, Jeollanam-do 519-763, South Korea; 2 Department of Pathology, Chonnam National University Research Institute of Medical Sciences, Chonnam National University Hwasun Hospital & Medical School, Hwasun-gun, Jeollanam-do, South Korea; 3 Department of Radiology, Chonnam National University Research Institute of Medical Sciences, Chonnam National University Hwasun Hospital & Medical School, Hwasun-gun, Jeollanam-do, South Korea

**Keywords:** Cavernous hemangioma, Complication, Outcome, Surgery, Third ventricle

## Abstract

**Background:**

Although cavernous hemangiomas (CHs) can be found anywhere in the central nervous system, CHs of the third ventricle have been reported in only 29 patients (including our case). In the current case report, we discuss the clinical characteristics and surgical outcome of CHs of the third ventricle.

**Case presentation:**

A 64-year-old female was admitted to our emergency room with a sudden decreased level of consciousness. Brain imaging studies demonstrated a multi-lobulated hemorrhagic mass in the third ventricle. The lesion was removed via the transcallosal-interforniceal approach and pathologically diagnosed as CH. Postoperatively, the patient had a transient neurological deficit due to hypothalamic injury and recovered to the normal status at 2 months after the operation. In the review of 29 cases, the mean age of the patients was 40 years with a slight female preponderance (female/male, 17/12). The majority of the patients complained of a mass effect with signs of increased intracranial pressure; only one case was asymptomatic. Gross total resection was achieved in 81% of the cases. Around 80% of the patients were asymptomatic or improved from the initial symptoms. Mortality rate was 6.9% and the most common complication was hydrocephalus.

**Conclusions:**

As demonstrated in the review of the previous reports, the outcome is favorable after surgical excision for CH of the third ventricle. Hence, surgical excision appears to be the treatment of choice for CH located in the third ventricle, which tends to grow rapidly resulting in a mass effect.

## Background

Cavernous hemangiomas (cavernomas, cavernous angiomas, cavernous malformations; CH) are vascular hamartomas that are reported to be found at any location in the central nervous system (CNS). Due to the increased use of computerized tomography (CT) scan and magnetic resonance imaging (MRI), more CHs have been diagnosed in recent years. However, intraventricular location of CHs is uncommon, and the incidence of intraventricular CHs has been reported to be only about 2.5 to 10.8% of all intracranial CHs [1,2]. The most frequent location of intraventricular CHs is the lateral ventricle and involvement of the third ventricle is quite rare. Based on the review of Medline database (PubMed, http://www.ncbi.nlm.nih.gov/PubMed), only 29 cases (including our case) of CH of the third ventricle have been reported [3-20].

We present the case of a patient who had a CH in the third ventricle that was resected through the transcallosal interforniceal approach. In addition, we also review the previously reported cases and discuss their clinical characteristics and surgical outcomes.

## Case presentation

**Figure 1:**
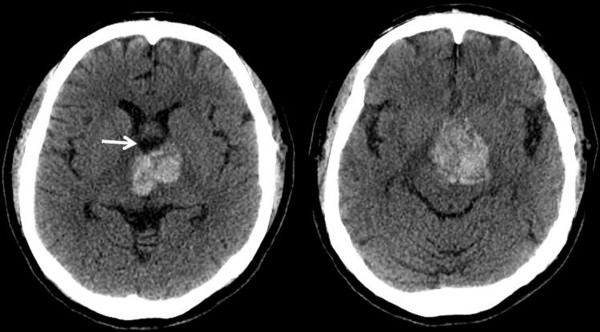
Axial non-contrast CT images show a large and heterogeneously hyperattenuated hemorrhagic mass in the suprasellar area, with dilatation of the anterior part of the third ventricle (arrow).

**Figure 2:**
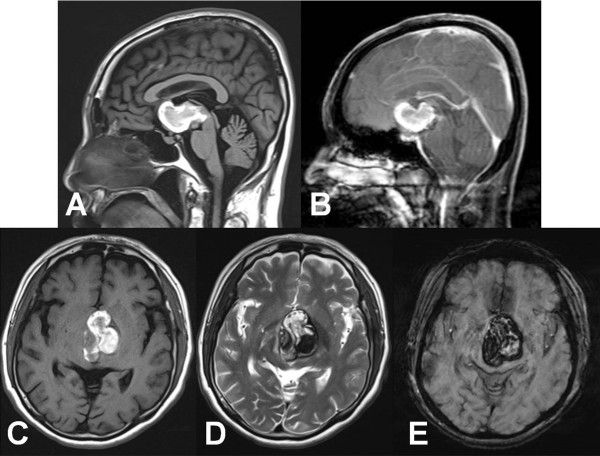
**Preoperative MRI images.** Sagittal T_1_-weighted **(A)** and post-contrast T_1_-weighted **(B)** images show a non-enhancing hemorrhagic mass in the anterior third ventricle and hypothalamic area. Axial T_1_-weighted **(C)**, T_2_-weighted **(D)**, and susceptibility weighted **(E)** images demonstrate a typical cavernous malformation with heterogeneous signal intensity and hemosiderin rim indicating mixed acute or subacute stage hemorrhage.

**Figure 3:**
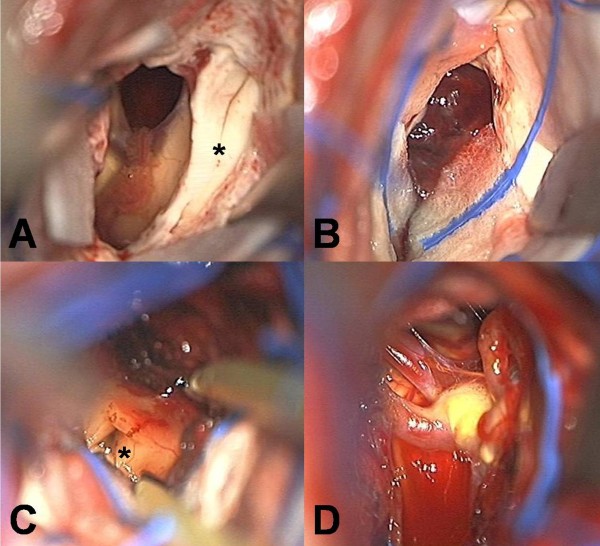
**Intraoperative photographs through transcallosal interforniceal approach. (A)** After dissection of the corpus callosum, the interforniceal plane (asterisk) was observed between the bilateral septum pallucidum; **(B)** After entering the third ventricle via the interforniceal approach, a red-colored and multi-lobulated cavernous hemangioma with numerous vascular channels and different-staged hemorrhage was encountered; **(C and D)** Removal of the lesion revealed the patency of the Sylvian aqueduct (asterisk) over the massa intermedia and the basilar system in the base of the lesion.

**Figure 4:**
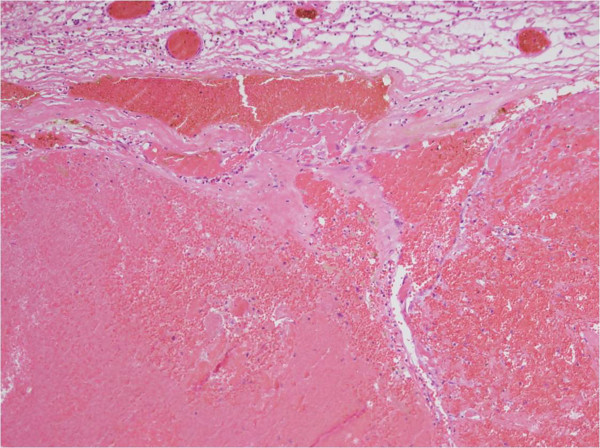
**Pathologic findings of the cavernous hemangioma.** The microphotograph displayed a blood-filled lesion composed of irregularly dilated channels and a slightly fibrotic capsule surrounding the lesion (hematoxylin and eosin, original magnification × 100).

A 64-year-old female was admitted to our emergency room with a sudden decreased level of consciousness. Except for an intermittent and mild degree headache, there was no specific history of head trauma and medical illness. On neurological examination, she showed a drowsy mentality with Glasgow Coma Scale score of 14/15 and the right homonymous hemianopsia. She did not have motor/sensory and cranial nerve deficits, and cerebellar signs. There were no abnormal laboratory findings. Non-contrasted CT scan showed a heterogeneously hyperattenuated hemorrhagic mass within the third ventricle compressing the hypothalamus, without definitive hydrocephalus (Figure 1). Brain MRI revealed a 40 × 30 × 28 mm sized multi-lobulated mass with a recent hemorrhage in the third ventricle, which extended to the foramen of Monro and hypothalamus. There was no definite contrast enhancement (Figure 2).Right-side interhemispheric, transcallosal interforniceal approach was used for removal of the lesion. At surgery, the lesion was found to be a red colored, multi-lobulated mass, which had numerous vascular channels and multi-staged hemorrhage. Although there were severe adhesions between the base of the lesion and the basilar arterial system, gross total removal of the lesion was possible due to the presence of the discrete sticky hemosiderin rim, which allowed differentiation of the lesion from the surrounded normal parenchyma (Figure 3). To prevent hypothalamic injury, the resection of hemosiderin-stained tissue was restricted to the minimum.Histopathological examination of the lesion revealed a CH composed of large, irregularly dilated, blood-filled vascular channels lined by flat endothelium (Figure 4). Postoperatively, the patient developed transient diabetes insipidus, somnolence, and general weakness due to hypothalamic injury, but these symptoms gradually disappeared with conservative treatment. Finally, she recovered to the normal status at 2 months after the operation.

## Discussion

CHs are vascular hamartomas which are reported to be found anywhere in the CNS. However, intraventricular CHs are rare and their incidence was reported to be only about 2.5 to 10.8% of all cerebral cavernous malformations [1,2]. CHs may be diagnosed based on symptoms of acute hemorrhage, seizures, or progressive neurologic deficits. Chadduck et al. [21] reported that there was no difference between the clinical symptoms and signs of intraventricular CHs and parenchymal CHs. However, because of the rarity, the natural history and clinical features of CHs located in the third ventricle have not been fully investigated and there are no definite recommendations for its management.

Overall, 29 patients with a well-described CH in the third ventricle have been reported in the literature, including our case [3-20] (Table 1), with a slight female preponderance (female/male ratio, 58/42%). The median age of the patients was 40 years (range, 8–64 years) and 6 patients were of the pediatric age group (21%). The most frequent initial clinical symptoms included a mass effect, with signs of increased intracranial pressure (headache, nausea, vomiting, visual disturbance, memory impairment and signs of hypothalamic dysfunction) in 23 patients (79%). Intraventricular hemorrhage from the lesion occurred in 3 cases (10%) and seizures in 2 cases (7%); only one patient was asymptomatic. This higher incidence of mass effect symptoms may be because of the direct compression of the surrounding structures, due to CH growth. Katayama et al. [5] stated that intraventricular CHs tend to grow rapidly resulting in giant malformation, because of low mechanical resistance caused by lack of the surrounding brain tissue and repeated hemorrhage in the CH. In the literature, the mean size of the lesions was reported to be 23 mm (range, 12–40 mm). Although intralesional bleeding can frequently occur when CHs grow within the ventricle, bleeding from a CH into the ventricular system is rare as per the previous reports [10].

**Table 1:** Summarized surgically resected cavernous hemangioma of the third ventricle

**Authors**	**Publication year**	**Age (year)**	**Sex**	**Symptom**	**Size (cm)**	**Approach**	**Extent of resection**	**Outcome**	**Postoperative complication**
Vaquero et al. [3]	1980	18	F	Diplopia	–	TC	GTR	Improved	
Pozzati et al. [4]	1980	31	F	Headache, vomiting	–	TV	GTR	Improved	
Lavyne et al. [5]	1983	48	F	Headache, memory impairment	1.5	TC + TV + SC	PR	Not improved	HDC, IVH
Amagasa et al. [6]	1984	40	M	Homonymous hemianopsia, endocrine function deficit	–	IH + TLT	GTR	Improved	
Harbaugh et al. [7]	1984	44	F	Headache, vomiting, IVH	2	TC + TV	GTR	Improved	HDC
Yamasaki et al. [8]	1986	9	M	Headache	2.5	–	GTR	Improved	
		15	F	Lower temporal quadrantopsia	1.5	–	PR	No symptom	
		36	M	Headache, vomiting, mental change	2.5	–	PR	Improved	
Voci et al. [9]	1989	19	F	IVH	–	TC	GTR	Improved	
Ogawa et al. [10]	1990	16	M	Headache, nausea	2	IH + TLT	GTR	No symptom	
		40	M	Homonymous hemianopsia, endocrine function deficit	2	IH + TLT	GTR	Improved	
Katayama et al. [11]	1994	9	F	Seizure	–	IH + TLT	PR	Death	
		50	F	–	–	–	–	Improved	
		45	F	IVH	–	–	–	Not improved	Vegetative state
		49	M	Visual field defect, endocrine function deficit	2	–	–	Improved	
		47	F	Memory impairment	3	SC + TVI	GTR	Improved	Transient DI, Recurrence
Sinson et al. [12]	1995	43	F	Headache, memory impairment	3	IH + TC + IF	GTR	Death	
		36	F	Memory impairment, weight gain	3	IH + TC + IF	GTR	Not improved	HDC
		52	F	Headache, nausea	3.5	TCo	GTR	Improved	
		32	F	Headache, vomiting, diplopia	2	IFT + SCbll	GTR	Improved	
Reyns et al. [13]	1999	42	M	Seizure	2.5	TCo + TVI	PR	Improved	Recurrence
Crivell et al. [14]	2002	38	M	Memory impairment, gait disturbance, headache, vomiting	–	TCo + TVI	GTR	Improved	
Wang et al. [15]	2003	62	F	Gait disturbance	–	TCo + TV	GTR	Not improved	ICH on thalamus, CNS infection
Milenkovic et al. [16]	2005	56	M	Headache, memory impairment, bizarre behavior	–	TC + TV + TF	GTR	Improved	
Darwish et al. [17]	2005	47	F	No symptom	1.5	TC + TV + TF	GTR	No symptom	HDC
Longatti et al. [18]	2006	35	M	Headache, vomiting, neck pain	1.2	TV	GTR	Improved	
Zakaria et al. [19]	2006	8	M	Headache, vomiting, gait disturbance	–	TC	GTR	Improved	
Kivelev et al. [20]	2010	52	M	Headache, vomiting	–	TC + IF(?)	GTR	Improved	
Present study	2012	64	F	Mental change, homonymous hemianopsia	4	TC + IF	GTR	Improved	Transient DI & hypothalamic injury symptoms*

*; totally resolved at 2 months after the operation.

–, not available; DI, Diabetes insipidus; F, Female; GTR, Gross total resection; HDC, Hydrocephalus; ICH, Intracerebral hemorrhage; IF, Interforniceal; IH, Interhemisphric; IFT, Infratentorial; IVH, Intraventricular hemorrhage; M, Male; PR, partial resection; SC, Subchoroidal; SCbll, Supracerebellar; TC, Transcallosal; TCo, Transcortical; TF, Transforaminal; TLT, Translamina terminalis; TV, Transventricular; TVI, transvelum interpositum.

The radiological findings of the intraventricular CHs do not differ from those of the intraparenchymal type [10]. Generally, on CT scans, the CH is suggested by the presence of a high density area, absence of perilesional edema, and mild or no contrast enhancement because of blood pool effects, calcification, and recent hemorrhage [22]. On MRI images, the CHs usually have mixed signal intensities. High signal intensities correlate with the presence of methemoglobin and low signal intensities correlate with calcifications and fibrosis within the lesion on T1- and T2-weighted images. A peripheral rim of low signal intensity correlates with the paramagnetic effect of hemosiderin [23].

A conservative treatment is appropriate for an asymptomatic CH located in the supratentorial parenchyma. However, CHs located in the third ventricle, surrounded by vital structures, are especially dangerous. It has also been documented that these lesions show a rapid growth [5], resulting in significant morbidity. For these reasons, the third ventricular CH needs to be treated more aggressively. As shown in Table 1, 80% of the patients were asymptomatic or improved from their initial symptoms after the surgical procedure. The most frequent post-operative complication was a hydrocephalus, observed in four patients. Postoperative mortality was 6.9% (2/29). The important point to be noted, as illustrated by our case, is that large-sized lesions frequently involve the hypothalamus [6,10,11]. Therefore, careful dissection of the lesion should be performed to prevent damage to the hypothalamus. To reduce this complication, minimizing the resection of hemosiderin-stained tissue and preservation of associated developmental venous anomalies are the key points, as in surgery for CHs located in the brain stem or cranial nerves [24,25]. Furthermore, during the operation for CHs buried in the parenchyma with a critical neurological function, initial dissection and removal of the lesion should be attempted on the short trajectory after observation of the surface changes caused by the hemorrhage [24,26]. Considering these principles, transcallosal-interforniceal approach can provide a direct, short corridor to the third ventricle with wide exposure of the lesion.

## Conclusions

Surgical excision appears to be the treatment of choice for CHs located in the third ventricle, which tend to grow rapidly and cause a mass effect. Using the short corridor to the third ventricle, obtaining wide exposure of the lesion, and minimizing resection of the surrounding hemosiderin-stained tissue can lead to a favorable surgical outcome, as demonstrated in the previous reports, including this report.

## Consent

Written informed consent was obtained from the patient for publication of this Case report and any accompanying images. A copy of the written consent is available for review by the Editor-in-Chief of this journal.

## Abbreviations

CH: Cavernous hemangioma; CNS: Central nervous system; CT: Computed tomography; MRI: Magnetic resonance imaging.

## Competing interests

The authors declare that they have no competing interests.

## Authors’ contributions

MSH and KSM drafted manuscript. KHL and SKL revised manuscript critically for important intellectually content. KHL and KSM helped acquisition and interpretation of data. KHL and SKL participated in reviewing literature and helped in conception and design of the study. KSM and SJ conceived the study and participated in its design and coordination. All authors read and approved the final manuscript.
